# A Sensitive Piezoresistive Tactile Sensor Combining Two Microstructures

**DOI:** 10.3390/nano9050779

**Published:** 2019-05-21

**Authors:** Xuguang Sun, Jianhai Sun, Shuaikang Zheng, Chunkai Wang, Wenshuo Tan, Jingong Zhang, Chunxiu Liu, Chang Liu, Tong Li, Zhimei Qi, Ning Xue

**Affiliations:** 1 State Key Laboratory of Transducer Technology, Institute of Electronics Chinese Academy of Sciences (IECAS), Beijing 100190, China; sunxuguang16@mails.ucas.ac.cn (X.S.); jhsun@mail.ie.ac.cn (J.S.); zhengshuaikang18@mails.ucas.ac.cn (S.Z.); wangchunkai16@mails.ucas.ac.cn (C.W.); tanwenshuo17@mails.ucas.ac.cn (W.T.); cxliu@mail.ie.ac.cn (C.L.); changliu8888@gmail.com (C.L.); tli@mail.ie.ac.cn (T.L.); zhimei-qi@mail.ie.ac.cn (Z.Q.); 2 School of Electronic, Electrical, and Communication Engineering, University of Chinese Academy of Sciences (UCAS), Beijing 100049, China; 3 School of Microelectronics, University of Science and Technology of China (USTC), Hefei 230026, China; zhang23@mail.ustc.edu.cn

**Keywords:** flexible electronics, tactile sensors, nanocomposite, electronic skin, microstructure

## Abstract

A tactile sensor is an indispensable component for electronic skin, mimicking the sensing function of organism skin. Various sensing materials and microstructures have been adopted in the fabrication of tactile sensors. Herein, we propose a highly sensitive flexible tactile sensor composed of nanocomposites with pyramid and irregularly rough microstructures and implement a comparison of piezoresistive properties of nanocomposites with varying weight proportions of multi-wall nanotubes and carbon black particles. In addition to the simple and low-cost fabrication method, the tactile sensor can reach high sensitivity of 3.2 kPa^−1^ in the range of <1 kPa and fast dynamic response of 217 ms (loading) and 81 ms (recovery) at 40 kPa pressure. Moreover, body movement monitoring applications have been carried out utilizing the flexible tactile sensor. A sound monitoring application further indicates the potential for applications in electronic skin, human–computer interaction, and physiological detection.

## 1. Introduction

Bionic electronic skin has attracted much research attention because of its great potential in medical treatment [1], human–computer interaction [2], robotics [3], and visual display [4]. Flexible tactile sensors play an important role in research of artificial skin. Based on the study of the perception mechanism of human skin for external pressure [5], good flexibility and high sensitivity are necessary characteristics for artificial tactile pressure sensors to achieve the desired sensing effect. In order to achieve this goal, various processes and materials are utilized in the manufacturing process. According to the working principle, tactile sensors are divided into piezoresistive [6], piezoelectric [7], capacitive [8], triboelectric [9], optical [10], and electromagnetic [11]. Among them, piezoresistive tactile sensors have attracted much attention because of their simple fabrication process, good robustness, and stability. Contact pressure can be perceived according to changes of sensor element resistances. In piezoresistive sensing elements, conductive paths are realized by various nanoscale conductive materials including carbon nanotubes (CNTs) [12,13], graphene [14,15], and carbon black (CB) [16], and the spatial structure of piezoresistive elements also determines the sensitivity of the sensor. For instance, Zhang et al. [17] proposed a highly sensitive flexible tactile sensor with three-axis force sensing capacity by combining the microstructured pyramids’ polydimethylsiloxane (PDMS) arrays and reduced graphene oxide (rGO) film. The deformation of microstructured rGO/PDMS results in the change of contact area between the rGO film and electrode, and the adoption of the pyramid structure increases the sensitivity of the sensor. Li et al. [18] developed a type of high-performance flexible capacitive tactile sensor utilizing bionic microstructures on natural lotus leaves. Taking advantage of the unique surface micro-pattern of lotus leaves as the template for electrodes and using polystyrene microspheres as the dielectric layer, the proposed devices present stable and high sensing performance. Wang et al. [12] presented a simple and low-cost method for fabrication of a large-area tactile sensor with patterned PDMS conducting thin films. By using the microscale surface texture of silk as the mold, they fabricated micro-patterned PDMS thin film and the sensing device demonstrated superior sensitivity. Chun et al. [19] suggested a highly sensitive tactile sensor using a conductive polyurethane sponge where graphene flakes were self-assembled into the porous structure of the sponge. The special porous structure in sponge provides a conductive path for the piezoresistive element and increases the sensitivity of the sensor at the same time. Nevertheless, there are still two major drawbacks for these tactile sensors. The first is that sensors with a high micro-pressure measurement limit hardly possess a large detection range, which results in saturation of the sensing device under a large tactile pressure and limits the application scenario. Another disadvantage is that high-sensitivity sensors are often accompanied by complex and high-cost fabrication processes, which will limit the large-scale application of sensors and introduce more uncertainties in the fabrication process. Therefore, a method that can balance a large sensing range, high sensitivity, and cost of fabrication needs to be proposed to make the tactile sensor have good comprehensive characteristics including large-scale fabrication capacity, high sensitivity, and good flexibility.

Herein, we propose a highly sensitive flexible tactile sensor by combining two types of microstructures in a stack structure with a simple and low-cost fabrication method. A pyramid structure and a double-sided rough structure are utilized to improve the sensitivity of the sensor and comparison of different structure distributions are implemented. Multi-walled carbon nanotubes (MWCNTs) and carbon black (CB) are filled into polydimethylsiloxane (PDMS) matrix as nanoscale conductive materials to obtain nanocomposites, and systematic comparison on proportions of different conductive materials and the piezoresistive property of a sensing device are carried out to determine the optimum filling ratio of CNTs. The tactile sensor in this work possesses good reliability, flexibility, a simple fabrication process, and shows short response times even under large contact pressure. What is more, the tactile sensor can detect muscle movement and help recognize different words from output signals by attaching to the human throat, which has the potential of language recognition.

## 2. Materials and Methods

**Figure 1:**
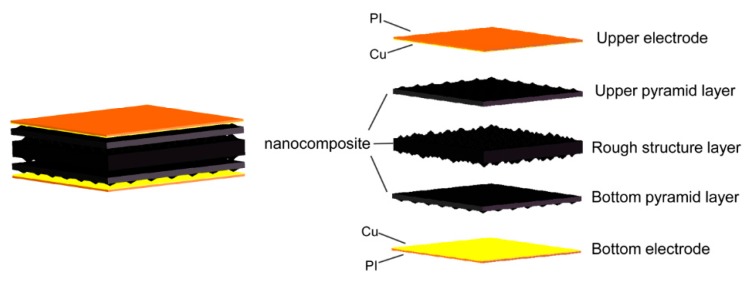
The schematic diagram of the decomposition structure of the tactile sensing device.

The electrode structure and relative distribution position of a piezoresistive tactile sensor represents the connection mode of the conductive path in the sensor. Different from interdigital electrodes in the same plane, electrodes in a face to face structure keep the tactile sensor negative resistance variation under compressive stress. As shown in Figure 1, the proposed tactile sensor is formed based on a sandwich structure. Two types of microstructural sensing layers (rough porous structure layer and pyramid layer) are sandwiched between the upper and bottom electrodes. The double-sided rough porous structure is sandwiched between the upper pyramid layer and bottom pyramid layer. Multi-microstructured layers enhance the sensitivity of the sensor and the rough structure layer possesses a rough irregular structure on both surfaces. The irregularly rough structure also increases the friction between the rough layer and the pyramid layers to avoid sliding displacement among the multi layers. Pyramid layers and the rough structure layer have identical nanocomposites consisting of PDMS and nanoscale conductive materials. The PDMS matrix ensures the structure layer has good flexibility and elasticity. The upper and bottom electrode layers are covered by a flexible polyimide (PI) film which can provide protection for the sensor and Cu electrodes. When a compressive load is applied on the sensor, the increased contact area among each structure and electrode layer and the constringent nanocomposite results in a lower resistance. As the load is removed, the structure layer can quickly restore its original shape.

### 2.1. Nanocomposite

**Figure 2:**
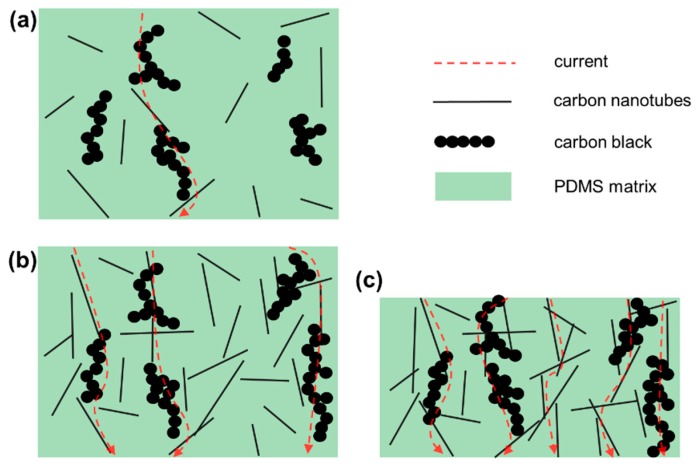
Schematic diagram of nanomaterial distribution in a polydimethylsiloxane (PDMS) polymer matrix. (**a**) Conductive networks in the nanocomposite at a low filling concentration; (**b**) conductive networks in the nanocomposite at a high filling concentration; (**c**) conductive networks in a high filling concentration nanocomposite under compressive strain.

The resistance of the nanocomposite changes as the loading pressure varies. The microstructured nanocomposite in the tactile sensor transforms external pressure information into electrical information and the composition of nanocomposites determines characteristics like sensitivity of tactile sensors to a certain extent. We utilized multi-walled carbon nanotubes (MWCNTs) and carbon black (CB) as the contents of nanocomposites, and the effects of different filling concentrations and relative proportions on piezoresistive properties of nanocomposites were compared. Figure 2 illustrates the distribution mode of MWCNTs and CB in the PDMS polymer matrix. MWCNTs have a very high aspect ratio and good conductivity, whereas the chain-like conductive carbon black particles have large surface area. The contact between MWCNTs and CB particles results in complex conductive pathways in nanocomposites. As shown in Figure 2a,b, with the increase of the concentration of MWCNTs and CB, the conductive paths in the nanocomposite increase and the resistance decreases. What is more, the compression deformation of the nanocomposite under external pressure will result in more contact between MWCNTs and CB particles, as shown in Figure 2c, thus generating more conductive paths and reducing the resistance. The high strength of MWCNTs can keep them with stable mechanical and electronical properties under external forces thus making the sensing device exhibit stable piezoresistive characteristics.

**Figure 3:**
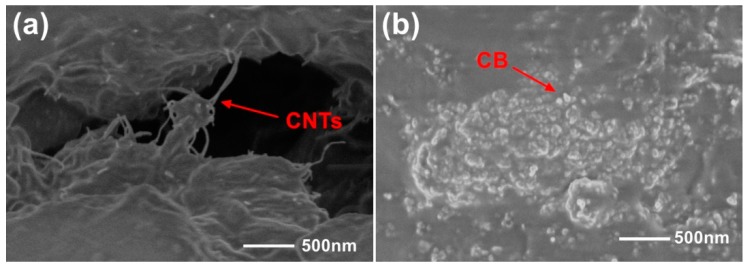
SEM images of internal of nanocomposites. (**a**) Multi-walled carbon nanotubes (MWCNTs) in the nanocomposite; (**b**) carbon black (CB) in the nanocomposite.

**Figure 4:**
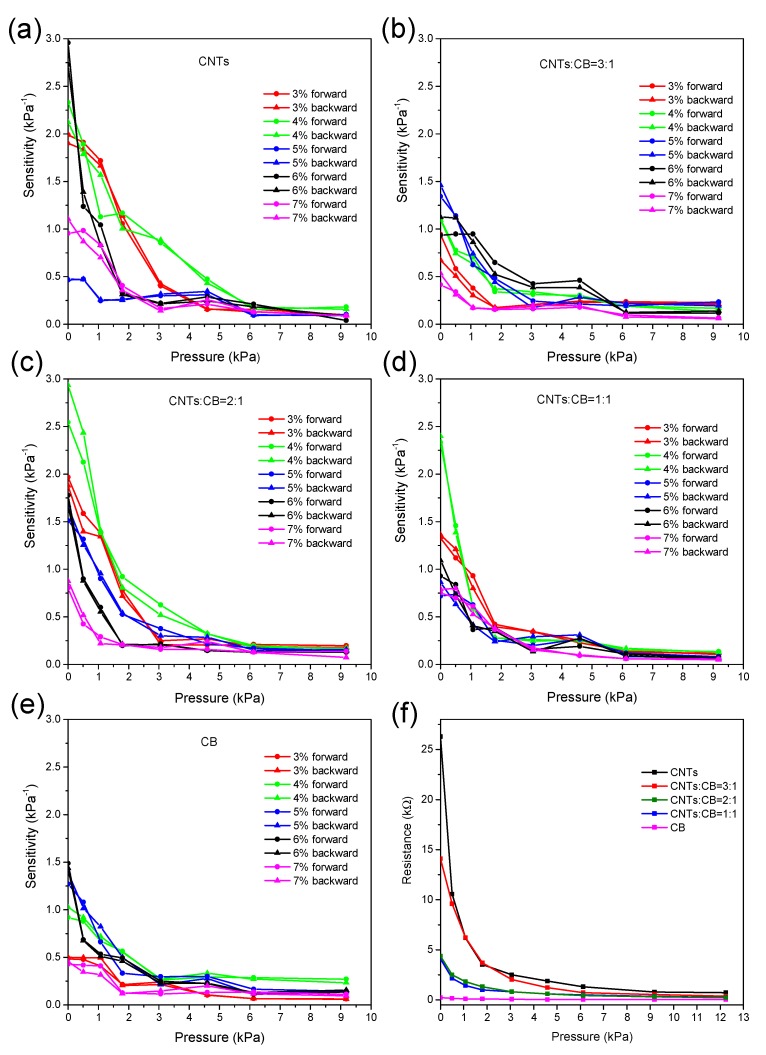
Sensitivity curves of the nanocomposite with different proportions of MWCNTs and CB. (**a**) MWCNTs only; (**b**) CNTs: CB = 3:1; (**c**) CNTs: CB = 2:1; (**d**) CNTs: CB =1:1; (**e**) CB only; (**f**) resistance curve of the nanocomposite element at 6% CNT content.

In our work, the synthesis of nanocomposites was made by a solution mixing and solvent evaporation method. Hydroxyl modified MWCNTs (purity >98%, −OH 5.58 wt %, Chengdu Organic Chemicals Co. Ltd., Chengdu, China) which have a good solubility without high aggregation in polymer solvents or CB (EC600JD, LION Co, Tokyo, Japan) and PDMS (Sylgard 184, Dow Corning Co, Midland, MI, USA) with a certain mass ratio were poured into a suitable amount of chloroform solution, respectively, and were mixed thoroughly. Then the two solutions were mixed together and phenylmethylsiloxane (PPMS, Alfa Aesar (China) Inc., Shanghai, China) was added into the solution at a mass ratio of 1:10 (PPMS/PDMS) to actualize better dispersibility of nanomaterials in the PDMS matrix. After that, the mixed solution was sonicated in an ultrasonic bath at 70 °C till the chloroform solvent was completely evaporated. Then the curing agent (1/10 of the mass of PDMS) was added into the nanocomposite with adequate agitation. Finally, the nanocomposite was placed in a vacuum chamber to extract internal gases. After curing at high temperature, the nanocomposite exhibits piezoresistive property and basically maintains the mechanical property of the PDMS matrix. Figure 3 illustrates scanning electron microscopic (SEM) details of MWCNTs and CB inside the nanocomposite. It can be seen that the MWCNTs are intertwined and contacted in composites to form intricate and uniformly distributed conductive networks. The CB particles uniformly distributed in the PDMS matrix can easily contact with each other to form conductive networks due to their large surface area. The basic number of conductive networks and the difficulty of increasing conductive networks are variable for nanomaterials with different proportions and contents in the PDMS matrix. The number of conductive networks determines the resistance, and the trend to alter the conductive networks matters to the sensitivity of the nanocomposite to a certain extent. In the next stage, comparison of the piezoresistive property of the sensing device on different proportions of MWCNTs and CB was carried out and the sensitivity curves are displayed in Figure 4.

Figure 4a–e illustrates the sensitivity curves of sensing elements composed of nanocomposites with different proportions of MWCNTs and CB, respectively. The nanocomposites in the sensing elements were made with a 200 μm thick, one-side rough structure with a size of 4 × 4 mm. The sensing elements with 3% to 7% CNT content have been measured with forward and backward pressure variations, respectively. Different pressures were applied to the sensor fixed on the platform by the pressure gauge and the resistance value of the sensor was displayed and recorded in real time by the multimeter. It can be seen that as the pressure increases, the sensitivity of the sensing elements with all proportions decrease. The sensing element filled solely by MWCNTs in the nanocomposite shows the highest sensitivity of more than 2.9 kPa^−1^ (2.9 times relative variation of resistance per kPa) in the range of <500 Pa at a content of 6%. With the increase of the CB ratio, the sensitivity of nanocomposite sensing elements tends to decrease and whereas the sensitivity of sensing elements with lower CNT content exhibits higher overall trends.

Compared with the nanocomposite solely filled by MWCNTs shown in Figure 4a, the sensitivity of the counterpart filled by CB shows obvious lower values. What is more, sensitivity measurement curves of the nanocomposite with different CNT contents coincide well, both forward and backward, which indicates good consistency and less hysteresis of the sensing device. Figure 4f displays the resistance variation of sensing elements with different proportions of MWCNTs and CB at 6% CNT content under external pressure. It is obvious that the sensitivity of CNT-filled nanocomposite is the highest and decreases significantly when the CB content increases.

Table 1 indicates initial resistance of piezoresistive sensing elements with different conductive nanomaterial proportions in nanocomposites and content of CNTs in mass. We can see that as the concentration of CNTs and CB increases, the initial resistance of conductive composites decreases because there is more interconnection and interpenetration among CNTs and CB particles contributing to more current paths in the nanocomposite. What is more, under the same content of CNTs, the initial resistance value of the piezoresistive sensing element decreases as the proportion of CB increases because carbon black particles can form more conducting channels for carriers between CNT chains, increasing the conductivity of piezoresistive materials.

### 2.2. Fabrication

**Figure 5:**
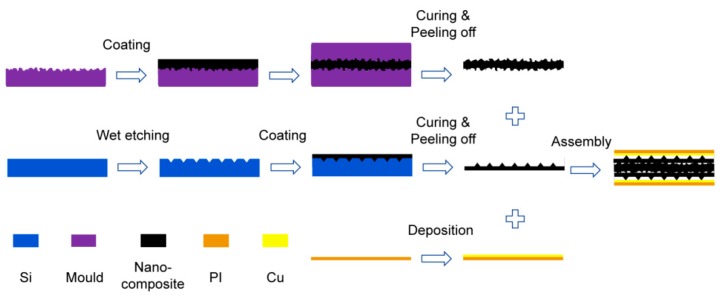
Fabrication process of the piezoresistive tactile sensor.

Figure 5 illustrates the fabrication process of the proposed tactile sensor. Based on the above analysis of piezoresistive characteristics of nanocomposites filled by different proportions of conductive materials, we use 6% MWCNT content nanocomposite as fabrication of the sensor. The main steps of fabrication include the following. First, a 200 μm thick abrasive paper with P400 grit degree was utilized as the mold and 30 μm thick prepared nanocomposite film was evenly knife coated onto the surface of the mold with a hard mask. Then another piece of the same abrasive paper was covered on the nanocomposite and the air inside the nanocomposite was exhausted in a vacuum drying chamber. After curing at 120 °C for 40 min and peeling off from the abrasive paper mold, the nanocomposite film was completely cured and possessed a double-sided rough surface structure. Second, 10 μm thick photoresist was spin-coated on a monocrystalline silicon substrate and square arrays were developed on the photoresist film by photolithography. The silicon wafer with patterned photoresist was wet etched in potassium hydroxide (KOH) solution to obtain the inverted pyramid structure, followed by 100 μm thick nanocomposite film coating on the inverted pyramid structure and curing at 120 °C for 40 min. Third, a 200 nm thick copper (Cu) layer was deposited on a 20 μm thick PI film to form the upper or bottom electrodes. After being peeled off from the abrasive paper mold and pyramid structure patterned substrate, the nanocomposite films with a double-sided rough structure and pyramid structure were cut into an 8 mm × 8 mm square and the fabrication of the tactile sensor was completed by assembling nanocomposite films and electrodes and fixing edges of the device by transparent polyurethane film tape, as shown in Figure 5.

## 3. Results and Discussion

**Figure 6:**
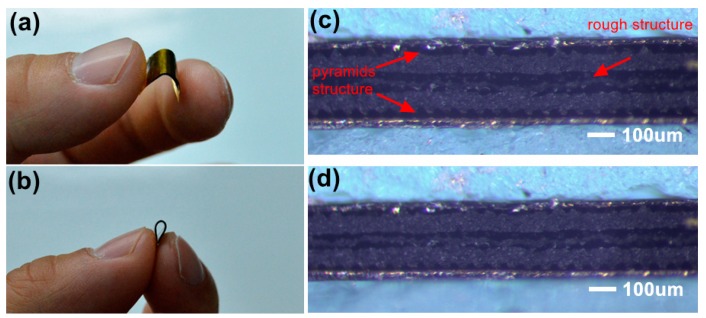
Optical images of the tactile sensor with (**a**) good flexibility showing (**b**) the bending limit. Optical images of the cross-section of the tactile sensor under (**c**) the initial state and (**d**) the compressive stress.

Figure 6a,b illustrates the flexibility of the tactile sensor. Fabricated from fully flexible material layers, the tactile sensor can bend at wide angles, revealing the ability of the sensor to attach on irregular surfaces in practical applications. The plane side of the pyramid structural layer contacts the middle rough structure layer, and the other pyramid structural side point contacts the electrode in the initial state, as shown in Figure 6c. When external pressure is applied to the sensor, the rough structure layer and pyramid layers suffer from compressive strain, seen in Figure 6d. The contact area between pyramids and the electrode surface and the contact area between the rough layer and the pyramid layer increase simultaneously. The resistance of the sensor can be written as:

(1) $$R=\frac{\rho \times l}{A}$$

where *ρ* is resistivity of the nanocomposite, *A* is the cross area between nanocomposite layers and electrodes, and *l* is the total length of nanocomposite layers. According to Equation (1), the increase of contact area and decrease of length of nanocomposite layers caused by compressive strain will lead to the decrease of resistance.

**Figure 7:**
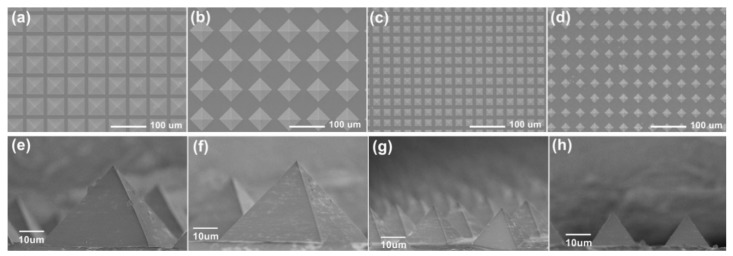
SEM photographs in top view and detail of the pyramid structure with different side lengths and intensive degrees. (**a**,**e**) 50 μm, dense; (**b**,**f**) 50 μm, sparse; (**c**,**g**) 20 μm, dense; (**d**,**h**) 20 μm, sparse.

In this work, the influence of different pyramid structures on the tactile sensor performance was compared and Figure 7 reveals the SEM images of different pyramid structures. Dense and sparse pyramid arrays with side lengths of 50 and 20 μm were fabricated, respectively. The spacing between adjacent pyramids in the dense array was 10 μm and the distribution density of the sparse array was half of that in the dense array, and individual pyramids in adjacent rows stagger with each other, which are shown in Figure 7b,d. The details in the SEM images illustrate good consistency and structural integrity of the pyramid structure.

**Figure 8:**
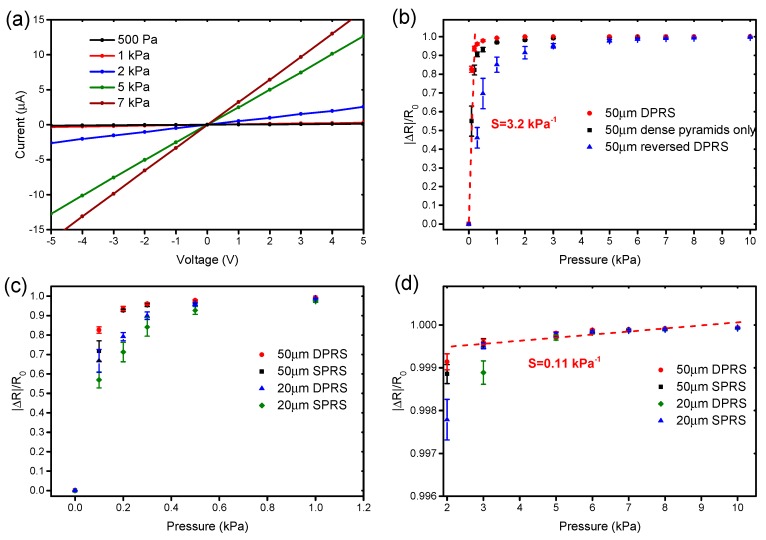
(**a**) Current-voltage curves of the tactile sensor under different pressures; (**b**) resistance variations of different structure layer combinations; resistance variations of different structure layer combinations in (**c**) the low pressure range and (**d**) the high pressure range. DPRS: dense pyramid structure and rough structure, SPRS: sparse pyramid structure and rough structure.

In addition to the comparison of different distributions of pyramid structure, different combination types of structure layers were also compared, and the current-voltage curves were measured, as shown in Figure 8a. The linear I–V curves reveal stable ohmic performance of the tactile sensor under varying pressures. Figure 8b shows the resistance variations of different combinations between pyramid structure layers with a 50 μm side length of the individual pyramid and rough structure layer. The combination types include DPRS (dense pyramid structure and rough structure), dense pyramids only (two dense pyramid layers contacted to each other directly back-to-back without a middle rough structure layer), and reversed DPRS (two pyramid layers placed in reverse to make the pyramid structure get interlocked with the rough structure). The results reveal that DPRS has the highest sensitivity of 3.2 kPa^−1^ in the pressure range of <1 kPa, and the dense pyramids only have the sensitivity of 3.0 kPa^−1^. As a contrast, the reversed DPRS has the lowest sensitivity of 1.53 kPa^−1^.

According to Equation (1), the change rate of resistance can be written as:

(2) $$∆R=\frac{∆\rho \times l}{A}+\frac{∆l\times \rho }{A}-\frac{∆A\times \rho \times l}{A^{2}}.$$

The definition of sensitivity is:

(3) $$S=\left|\frac{∆R/R}{∆P}\right|.$$

When the nanocomposite layer is subjected to compressive stress, the change of resistivity ∆ρ is negligible comparing to the change of sensor length. Additionally, the relative strain under unit pressure can be expressed as Young’s modulus by definition. Thus, the sensitivity of the tactile sensor can be reorganized as:

(4) $$S=\frac{\left|∆l\right|/l}{∆P}-\frac{\left|∆A\right|/A}{∆P}=\frac{1}{E}-\frac{\left|∆A\right|/A}{∆P}\;.$$

As displayed in Figure 8b, the resistance variation of the tactile sensor can be divided into two stages obviously. In the ultralow pressure range, the sensor shows pretty high sensitivity and the sensitivity decreases significantly as the pressure exceeds 1 kPa. To explain the phenomenon theoretically, the low pressure results in smaller overall elastic modulus of the tactile sensor as the deformation of the microstructure layers is relatively small. According to Equation (4), the sensitivity of the sensor at this stage becomes higher. With the increase of the compressive strain of nanocomposite structure layers, Young’s modulus E of the sensor increases, which results in a lower sensitivity. Figure 8c,d illustrates resistance variations of sensors with different pyramid structure layers in the low and high pressure range, respectively. It can be seen that the sensitivity of DPRS is higher than that of the sparse pyramid structure and rough structure (SPRS) with the same side length. Meanwhile, DPRS and SPRS with 50 μm side length possess higher sensitivity than DPRS and SPRS with 20 μm side length. In the pressure range exceeding 2 kPa, DPRS and SPRS with 50 μm side length show a similar sensitivity of 0.11 kPa^−1^.

The characteristic of high sensitivity in the low pressure range and low sensitivity in the high pressure range guarantees the tactile sensor suitable for practical application scenarios on simultaneous detection of small contact forces with high sensitivity and larger contact forces with unsaturated states in the large pressure range. Table 2 lists the comparison of this work with several recent reports. The test result indicates good compatibility and balance between pressure sensitivity and detection range, and good properties of our flexible tactile sensor.

**Figure 9:**
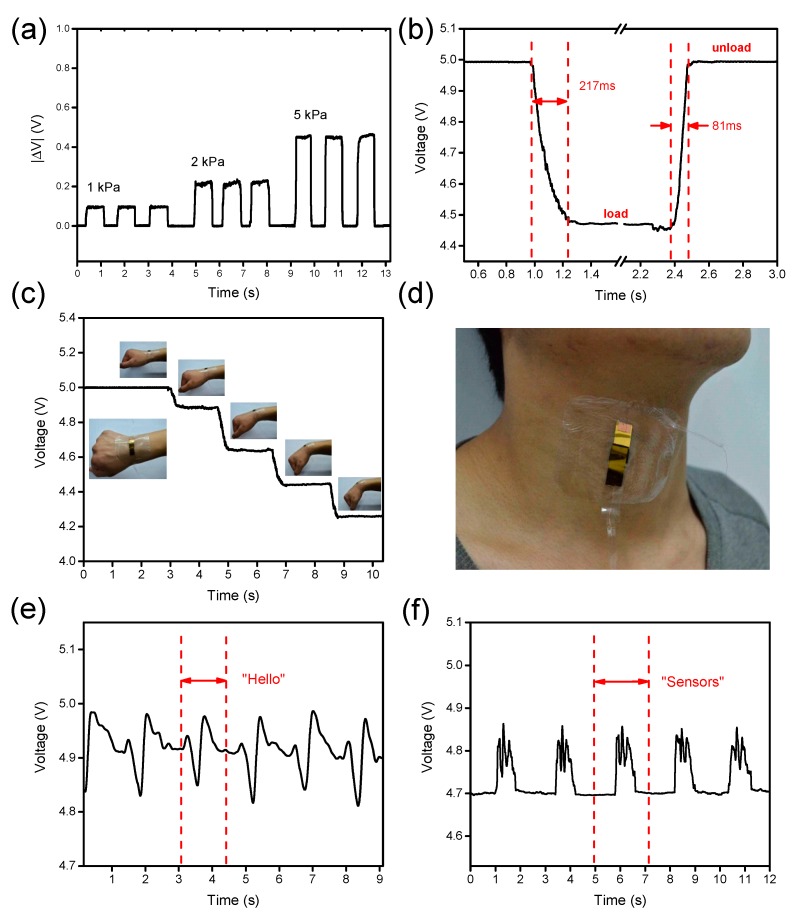
(**a**) Dynamic voltage variation under different pressures; (**b**) loading and unloading response times of the tactile sensor under 20 kPa pressure. (**c**) Detection for wrist bending with different angles. (**d**) Detection for muscle movement in speech. (**e**,**f**) Muscle movement signals monitored by the sensor when speaking a word repeatedly.

As shown in Figure 9a, the flexible tactile sensor performs with good response stability and repeatability under the dynamic pressure loading and unloading process. The corresponding pressure was applied to the sensor through the pressure gauge cyclically and the output voltage of the sensor in a constant voltage circuit was recorded by the oscilloscope in real time. Benefiting from the good elasticity of polymer in the nanocomposite, the tactile sensor device has a short response time even under ultrahigh pressure, which is displayed in Figure 9b. A 20 kPa pressure was applied to the sensor and released quickly, and the response time under 20 kPa was 217 ms and the recovery time was 81 ms. Figure 9c shows the resistance variation according to wrist bending in different angles. The resistances of the sensor element under bending angles of 5°, 15°, 30°, and 45° are 81, 23, 14.6, and 10.5 kΩ respectively. Figure 9d illustrates the image of application of monitoring human sounding by gently attaching the sensor to the neck near the throat. The real time monitoring of repeated signals of speaking a word are displayed in Figure 9e,f, respectively.

The high sensitivity, fast response time, good stability, and flexibility of the tactile sensor indicate wide applications in electronic skin and medical monitoring. The sensor shows good angle resolution in the measurement of wrist bending, providing the possibility of sensitively detecting the movement of human muscles and showing its potential application in human–computer interaction and physiological detection. The resistance of the tactile sensor changes steadily and consistently when the human body speaks different words. The detection of different human sounds demonstrates the potential of speech recognition of the sensor from the perspective of tactile perception and judging the voice status in real time by combining with a machine learning method.

## 4. Conclusions

In this work, a highly sensitive flexible tactile sensor was proposed by combining a pyramid structure and a double-sided rough structure with a simple and low-cost fabrication method. Based on the comparison results of the piezoresistance of nanocomposites with different proportions of MWCNTs and CB particles, 6% content of MWCNTs in the nanocomposite was chosen with the best performance. Moreover, different structure combinations were compared and the tactile sensor with 50 μm DPRS showed the highest sensitivity of 3.2 kPa^−1^ in the range of <1 kPa, and 0.11 kPa^−1^ exceeding 1 kPa. The tactile sensor possessed a short response time under a large pressure and has good repeatability. What is more, body movement monitoring applications were carried out which demonstrate great potential of the tactile sensor in applications of electronic skin, human–computer interaction, physiological detection, and sounding recognition.

## Figures and Tables

**Table 1:** Initial resistance of nanocomposites under different nanomaterial compositions and proportions. The unit is ohms (Ω).

-	3%	4%	5%	6%	7%
CNTs	3 M	2.2 M	40 k	25 k	2.1 k
CNTs/CB = 3:1	475 k	71 k	20 k	14 k	1 k
CNTs/CB = 2:1	105 k	27 k	5.3 k	4.1 k	330
CNTs/CB = 1:1	7 k	2.9 k	290	210	140
CB	526 k	156 k	5.8 k	4 k	300

**Table 2:** The comparison of the pressure response and sensing range between this work and the recent reported works.

Sensing Materials	Sensing Mechanism	Pressure Sensitivity	Detection Range	Ref.
Carbon nanofiber networks	Piezoresistive	1.41 kPa^−1^	4.5 kPa	[20]
Single-walled carbon nanotubes/PDMS	Piezoresistive	3.26 kPa^−1^	3 kPa	[13]
Nanoparticles	Capacitive	1.0 kPa^−1^	11 kPa	[21]
Conductive polymers	Piezoresistive	17 kPa^−1^	5 kPa	[22]
CNT-PDMS composite	Piezoresistive	15.1 kPa^−^^1^	59 kPa	[23]
Polystyrene microspheres	Capacitive	0.815 kPa^−1^	500 kPa	[18]
MWCNTs-PDMS nanocomposite	Piezoresistive	3.2 kPa^−^^1^	40 kPa	Our work
